# Subcellular Localization of ENS-1/ERNI in Chick Embryonic Stem Cells

**DOI:** 10.1371/journal.pone.0092039

**Published:** 2014-03-18

**Authors:** Sophie Blanc, Florence Ruggiero, Anne-Marie Birot, Hervé Acloque, Didier Décimo, Emmanuelle Lerat, Théophile Ohlmann, Jacques Samarut, Anne Mey

**Affiliations:** 1 Institut de Génomique Fonctionnelle de Lyon, Université de Lyon, Université Lyon 1, CNRS UMR 5242, INRA USC 1370, Ecole Normale Supérieure de Lyon, Lyon, France; 2 Laboratoire de Génétique Cellulaire-INRA, ENVT, Castanet Tolosan, France; 3 CIRI, International Center for Infectiology Research, Université de Lyon, INSERM U1111, Ecole Normale Supérieure de Lyon, Lyon, France; 4 Université de Lyon, Lyon, France; Université Lyon 1, Villeurbanne, France; CNRS, UMR5558, Laboratoire de Biométrie et Biologie Evolutive, Villeurbanne, France; Wellcome Trust Centre for Stem Cell Research, United Kingdom

## Abstract

The protein of retroviral origin ENS-1/ERNI plays a major role during neural plate development in chick embryos by controlling the activity of the epigenetic regulator HP1γ, but its function in the earlier developmental stages is still unknown. ENS-1/ERNI promoter activity is down-regulated upon differentiation but the resulting protein expression has never been examined. In this study, we present the results obtained with custom-made antibodies to gain further insights into ENS-1 protein expression in Chicken embryonic stem cells (CES) and during their differentiation. First, we show that ENS-1 controls the activity of HP1γ in CES and we examined the context of its interaction with HP1γ. By combining immunofluorescence and western blot analysis we show that ENS-1 is localized in the cytoplasm and in the nucleus, in agreement with its role on gene's promoter activity. During differentiation, ENS-1 decreases in the cytoplasm but not in the nucleus. More precisely, three distinct forms of the ENS-1 protein co-exist in the nucleus and are differently regulated during differentiation, revealing a new level of control of the protein ENS-1. In silico analysis of the Ens-1 gene copies and the sequence of their corresponding proteins indicate that this pattern is compatible with at least three potential regulation mechanisms, each accounting only partially. The results obtained with the anti-ENS-1 antibodies presented here reveal that the regulation of ENS-1 expression in CES is more complex than expected, providing new tracks to explore the integration of ENS-1 in CES cells regulatory networks.

## Introduction

Endogenous retroviruses (ERVs) are known to play an important role in the expression of their host genome, notably during the first developmental stages when totipotent [1] or pluripotent cells [2] adopt new cell fates. ERV's promoters act as enhancers in different cellular models and lineages [3]. More rarely, ERVs introduce coding sequences that are adopted by the host genome to play a major role in host survival. This is the case for Syncitin, involved in formation of the mammalian placenta [4], and for ERNI, also called ENS-1 [5], that controls the timing of the neural plate emergence during chick embryonic development [6]. More precisely, ENS-1/ERNI acts as a boundary element between the epigenetic regulator HP1γ and the protein complex that is recruited to the promoter of the neural plate inducer *Sox2* before its expression. This linker property involves two distinct motifs in the protein, the HP1box engaged with HP1γ and the coiled-coil domain interacting with other proteins recruited to the promoter. The repression, mediated by HP1γ, on the promoter of *Sox2* is released in the prospective neural plate due to competition between ERNI and the newly synthesized protein BERT, another coiled-coil domain protein that does not bind HP1 [7].

In addition, *Erni* is expressed earlier during the chick developmental process notably in the hypoblast, in the pluripotent epiblast [8] and in its derived embryonic stem cells, cultivated *in vitro* [9], where it was called *Ens-1*. Silencing of the gene occurs later, as final differentiation is achieved [8], [9]. In the epiblast, this expression pattern is managed by the pluripotency transcription factor Nanog and by a combination of Gata and Ets transcription factors that are expressed in the epiblast, in the hypoblast and in the prospective neural plate [8]. It is probable that the ERV *Ens-1/Erni* controls transcription of the host's genome in pluripotent cells either directly with its promoter sequences spread over the genome [5], or indirectly by controlling HP1γ on its target genes. Among them *Sox2* is also known as a key-player in the maintenance of pluripotent cells in mammals [10], [11], but other genes, mainly involved in cell proliferation control, have also been described in mouse ES cells as HP1γ target genes [12].

Despite its important role during chick development, demonstrated using transient transfections, the expression of the *Ens-1* gene has only been followed at the transcriptional level and the endogenous protein ENS-1 has never been observed.

In this study, we raised ENS-1 specific antibodies in order to follow ENS-1 expression in CES cells and during their differentiation. Our results reveal that the distribution of ENS-1 is a new level of its regulation.

## Materials and Methods

### Cell culture and DNA transfection

CES are pluripotent stem cells that were isolated from chick embryonic epiblast [13] and expressing the pluripotency supporting genes [14]. CES were cultivated as indicated previously [15]. Plasmid DNA transfections were performed using Lipofectamine 2000 (Invitrogen) in cell cultures at 80% confluency according to the manufacturer's instruction. Stable overexpression was obtained using linearized DNA plasmids, and stably transfected cells were selected by culture in medium supplemented with puromycin (200 μg/ml). COS7 cells were cultivated with DMEM supplemented with 6% fetal calf serum, 1% penicilin/streptomycin, 1% glutamin.

### Antibodies production

The anti-Ens-1 antibody used for immunofluorescence experiments was obtained by immunization of mice with the recombinant ENS-1 protein produced in *Escherichia coli* as follow. The full-length coding sequence of the ENS-1 gene was fused in C-terminal with a 6x-histidine Epitope tag in a pET-22 expression system and transformed in Rosetta (DE3)pLys Competent Cells (Novagen). Protein production was induced with 1 mM IPTG for 3 hours at 37°C and immediately checked by direct loading of cells on a SDS-polyacrylamide gel followed by Coomassie blue staining. Cells were then lysed by sonication and the different fractions (soluble and insoluble cell extracts) were kept for further analysis. As ENS-1 is predominantly detected in inclusion bodies, the insoluble fraction was isolated by centrifugation and solubilized in 8 M urea, 100 mM NaH_2_PO_4_, 10 mM Tris HCl (pH 8.0). ENS-1 His tag protein was purified on Ni-NTA columns (Qiagen) and refolded by overnight dialysis in 50 mM NaH_2_PO_4_, 300 mM NaCl, 10 mM Tris HCl (pH 8.0). Purification efficiency was checked by Coomassie blue staining of a SDS-polyacrylamide gel loaded with the purified ENS-1 protein before its use as immunogen for antibody production. Production of the monoclonal antibody was carried out by Covalab (France) and immunizations were performed using complete Freund adjuvant according to the relevant legislation and following protocols approved by the ethic committee of the University of Bourgogne (France). Antibody producing hybridomas were first selected by Covalab for their reactivity with ENS-1 coated wells in ELISA tests. Positive hybridomas were next screened for use in western-blotting and in immunostaining. Their reactivity was tested in CES cells and in cells devoid of the gene Ens-1 as negative control. The selected IgG (from clone 16h4g6e4) was purified on protein A-coupled to Sepharose and called 16h4 in the results part. This unique clone worked in immunostaining and its specificity is demonstrated in the results section.

As none of the monoclonal antibodies produced worked in western-blot, we developed polyclonal antibodies in rabbits using peptides of 16–20 amino acids isolated from the ENS-1 sequence as immunogens. Design and purification of the peptides, rabbit immunizations and antibody purification from the serum were carried out by Eurogentec using its proprietary adjuvant combination and following protocols approved by the ethic committee of the CER groupe (Belgium) according to the European legislation. One of the three tested peptides (named 3807: C-DRIRVLQNEARTRAGK-CONH_2_) gave a specific polyclonal antibody working in western blot as described in the results section. This antibody did not work in immuno-staining. The 3807 peptide (DRIRVLQNEARTRAGK) is localized upstream the coiled-coil domain in the N-terminal part of the protein and spans amino-acids in position 47 to 62 of the protein (GenBank: AAK06824.1).

### DNA constructs

For transient overexpression experiments, chick HP1γ and Ens-1 (Genbank: NM_001080873) were cloned in a pCi expression vector (Promega) modified to introduce a FLAG tag at the N-terminal part of the protein. HP1γ (Genbank: NM_204643) was amplified from chicken ES cDNA. For *in vitro* translation, Ens-1 was cloned in a pGBKT7 expression vector (Clontech). Genes expressed in fusion with a GFP protein at their N-terminal part were cloned in pEGFP-C1 vector (Clontech).

For stable overexpression, Ens-1 was cloned in a bicistronic construct (pCX Ens-1-HA-ires-puro) to express ENS-1 in fusion with two HA-tags at the C-terminal part of the protein. In this construct Ens-1 was separated from the puromycin resistance gene by an IRES, allowing translation of both proteins from a common mRNA transcribed under the control of a CAG promoter (a chicken β-actin promoter combined with a CMV enhancer) to ensure a strong expression [16].

To test the transcriptional activity of proteins using the CAT reporter assay, the genes were cloned in the pM vector (Clontech) to express HP1γ or ENS-1 in fusion with a Gal4 DNA binding domain at their N-terminal part. The reporter plasmid pG4-TK-CAT (Chloramphenicol Acetyl Transferase) contains six copies of the Gal4 binding sites upstream of a TK promoter to control CAT expression. pG4-TK-CAT was a gift from Dr E. Manet and has been described elsewhere [17]. The renilla luciferase reporter vector pRL-CMV, used as control, was from Promega.

Targeted mutations of ENS-1 were performed using the Quick Change Mutagenesis Kit (Stratagene) following the manufacturer's instructions. Mutation of the HP1 box (PxVxL) was performed using the following primers:

Sense:TTGATGAATGGATTAGCCACAGCCAGAGCCGAGAAATTAGTTAAC; antisense:GTTAACTAATTTCTCGGCTCTGGCTGTGGCTAATCCATTCATCAA. The control mutation was performed on a PxVxL motif that was not involved in the interaction of ENS-1 with HP1γ as assessed using a two-hybrid assay in yeast described previously [7]. The primers used were: 

sense:TTGATGAATGGATTAGCCACAGCCAGAGCCGAGAAATTAGTTAAC,

antisense: GTTAACTAATTTCTCGGCTCTGGCTGTGGCTAATCCATTCATCAA.

In both PxVxL mutants the proline, valine and leucine amino acids were replaced by an alanine.

Deletion of the coiled-coil domain from position 238 to position 411 of the Ens-1 coding sequence was obtained by PCR amplification in two fragments of the sequence flanking the coiled-coil domain. Primers were designed to introduce an EcoRI motif that was used to ligate both amplified fragments. The fragment upstream of the coiled-coil domain was amplified using the following primers:

sense:ACTCGAGCCACCATGAGCAACAGTATGGCC;

antisense:ACCGGTGTCCTTACACGTGAACTCCACAGCTGCTTT. To amplify the fragment downstream of the coiled-coil domain, the primers were: sense: ACGTGTAAGGACACCGGT; antisense: TACGCGTTCAGCTCCCCTTGAGCTT. The deletion of the coiled-coil domain lowers the molecular weight of the ENS-1 protein by 5.5 kDa.

To measure the activity of both start codons in the ENS1 5′ UTR, fragments were cloned downstream to the T7 promoter in the p0renilla vector previously described [18] as illustrated in the results. The human β-globin 5′-UTR with the authentic initiation codon was obtained by hybridizing two synthetic oligodeoxyribonucleotides (Eurogentec) downstream to the T7 promoter to generate the pGlobin-renilla vector used as control (CTR) [18].

### Pull-down experiments

Chicken HP1γ cloned in the pCi-flag plasmid was produced in COS7 cells upon transfection with Exgene 500 (Fermentas) following the manufacturer's instructions. The next day, lysis of COS7 cells was performed (lysis buffer: 50 mM Tris-HCl pH 7.4; 150 mM NaCl; 1 mM EDTA; 1%Triton X-100; 1X Complete protease inhibitor cocktail (Roche); 1X Halt phosphatase inhibitor cocktail (Thermo Scientific)), the lysate was centrifuged and the supernatant was incubated with [^35^S]-Met (Amersham) to label ENS-1 synthesized *in vitro* using a TnT Coupled Reticulocyte Lysate Systems kit (Promega) and pGBKT7-Ens1 plasmid. The mixture was incubated for 5 hours at 4°C on a rotating shaker. The flag-HP1γ protein was precipitated using Anti-flag M2 affinity gel (Sigma) incubated with the protein mixture overnight at 4°C on a rotating shaker. After centrifugation the agarose beads were washed four times in washing buffer (50 mM Tris-HCl pH 7.4; 150 mM NaCl). Bound proteins were eluted with loading buffer at 100°C and analyzed by SDS-PAGE and fluorography using a STORM Phosphorimager.

### Immunofluorescence labeling

Cells were seeded on glass cover slides previously coated with gelatin 1% in 24 wells plates and cultivated to reach 80% confluency. CES cells were seeded with irradiated feeder cells and cultivated on the cover slides 48 h before analysis except for differentiated CES. In this case differentiation was induced 24 h after seeding without feeder cells, and using 10^−6^M retinoic acid as previously described [8] for the indicated period of time. Culture medium was removed and fixation was performed with 2.5% paraformaldehyde in PBS for 20 min at room temperature. After washing tree times with PBS, the cells were saturated and permeabilized with saturation buffer (PBS, 0.1% Triton X-100, 10% fetal calf serum). Primary antibodies diluted in saturation buffer were incubated 1 h at room temperature. After washing three times with PBS, the secondary antibody diluted in saturation buffer was added for 1 h at room temperature. Following washing three times with PBS, cover slides were mounted on a slide using Gelmount (Biomeda) supplemented with Draq5 (Biostatus) diluted 1/1000. For co-localization studies, cells were incubated with a mixture of the anti-ENS1 monoclonal antibody 16h4 (5 μg/ml) produced in mice and the rabbit anti-human HP1γ antibody from Abcam (Ab10480, 1/200). After washing, the cells were incubated with both Alexa secondary antibodies (1/1000) simultaneously. F(ab')2 goat anti-mouse IgG conjugated with Alexa 488 or Alexa 555 or F(ab')2 goat anti-rabbit IgG conjugated with Alexa 555 were from Molecular Probes. In Fig. S1 immunostaining of CES was performed using the 42s2 mouse monoclonal antibody (Upstate) directed against HP1γ in addition to the Ab10480 (Abcam).

Acquisition of the images was performed with a Zeiss LSM510 Confocal microscope using a 63× (NA 1.4) Plan NeoFluor objective (PLATIM, UMS 3444 Biosciences Gerland-Lyon Sud). Each channel was imaged sequentially using the multi-track recording module before merging. Experiments were performed at least three times and gave similar results.

### Electron microscopy

CES cells dissociated with trypsin-EDTA (Gibco) were pelleted by centrifugation and fixed with PFA 4% in PBS for 30 min in ice. These fixative conditions decreased the detection of the ENS-1 protein observed by immunofluorescence but were required to preserve most of the cell structures upon the following treatment.

Cells were then washed in PBS, mixed with 1% low melting point agarose and aspirated into a syringe. After agarose solidification, cells embedded into agarose were extruded from the syringe and the resulting extruded rods were cut into 3–5 mm^3^ cylinders. Samples were washed in PBS at room temperature for 30 min, dehydrated in 70% ethanol and embedded in LR White resin (Electron Microscopy Sciences). Ultrathin sections were mounted on nickel grids and immunolabelling performed by floating the grids onto drops of the different solutions. We used PBS-1% BSA as saturation buffer. The anti-ENS-1 antibody (clone 16h4) and the anti-human androgen receptor antibody (441, Santa Cruz) used as IgG1 negative control were diluted in saturation buffer and incubated 1 h with the cells. After washing in PBS the cells were incubated for 1 h at room temperature with donkey anti-mouse IgG antibody conjugated with 6 nm diameter gold particles (EM grade, Jackson Immunoresearch) diluted in saturation buffer. After washing in PBS, the labeling was fixed with PBS-2% glutaraldehyde for 10 min, washed three times with PBS and once with water. Ultrathin sections were then contrasted with uranyl acetate and lead citrate, and examined with a Philips CM 120 electron microscope equipped with a Gatan Orius 200 2Kx2K digital camera (Centre Technique des Microstructures, Université Lyon 1, Villeurbanne, France).

### Protein extractions and western blot

Whole cell lysates were prepared using RIPA lysis buffer (1% Triton X-100; 0.5% Sodium deoxycholate; 0.1% SDS; 150 mM NaCl; 0.1 mM DTT; 1 mM EDTA; 1 mM N-ethylmaleimide, Complete proteases inhibitor (Roche) in Tris-HCl 50 mM, pH 7.4). Following dissociation using trypsin-EDTA and washing in PBS, cells were suspended in cold RIPA lysis buffer (40×10^6^ cells/ml) and incubated 30 min in ice. The lysate was centrifuged at 10000 *g* for 30 min at 4°C. The supernatant was harvested and protein concentration was measured using Bradford reagent (Sigma) and BSA in the standard curve. The indicated protein quantities were mixed with loading buffer (10% glycerol, 2% SDS, 0.7 M β-mercaptoethanol, 0.01% bromophenol blue in 62.5 mM Tris-HCl pH 6.8) and denaturation was performed 3 min at 100°C.

Fractionation experiments were performed using two protocols. In the first one, cells were disrupted using detergent before the cytoplasm and the nucleus were separated. Dissociated cells were suspended (60×10^6^/ml) in cold buffer A (340 mM sucrose, 60 mM KCl, 15 mM NaCl, 2 mM EDTA, 0.5 mM EGTA, 0.1 mM DTT, 0.65 mM spermidine, 0.1% Triton X-100, Complete EDTA (Roche), 10 mM N-ethylmaleimide in 15 mM Tris-HCl pH7.4) and incubated 1 min in ice. The cytoplasm and the nuclei were separated by centrifugation 1500 *g* for 5 min at 4°C. The cytoplasm corresponding to the supernatant was clarified from residual nuclei by centrifugation 13000 g at 4°C for 5 min and the supernatant was harvested (cytoplasmic fraction, C). The nuclei containing pellets were suspended in buffer A and washed by deposition on a buffer A cushion in a 14 ml falcon tube followed by centrifugation at 9400 g for 10 min at 4°C. The nuclei were disrupted using the hypotonic buffer B (2 mM EDTA, 0.5 mM EGTA, 0.1 mM DTT, Complete EDTA (Roche), 10 mM N-ethylmaleimide in water). After 30 min incubation in ice, nuclei disruption was checked using a microscope. Next the soluble and insoluble nuclei components were separated by centrifugation 18000 g for 5 min at 4°C. Protein concentration was measured using Bradford reagent (Sigma) in the cytoplasmic fraction and in the soluble fraction of the nucleus containing fractions and the indicated quantities were mixed with protein loading buffer. The insoluble nucleus fraction was suspended in buffer A and mixed with the loading buffer (N) without measurement of the concentration but the volume corresponding to 6×10^6^ cells was loaded to the gel.

In the second fractionation protocol the cells were disrupted without detergent according to the protocol provided by Abcam with some modifications. Briefly, cells were dissociated in PBS using a scraper, rapidly pelleted by centrifugation, re-suspended in cold fractionation buffer (250 mM sucrose, 20 mM Hepes, 10 mM KCl, 1.5 mM MgCl2, 1 mM EDTA, 1 mM EGTA, 1 mM DTT, 1 mM N-ethyl maleimide, Complete protease inhibitor (Roche)) and passed through a 26G needle before 30 min incubation on ice. Cell disruption was checked using a microscope. The nuclei were separated from the cytoplasm by centrifugation 1500 g for 5 min at 4°C. The supernatant contained the cytosol, the cytoplasmic membrane and mitochondria, the pellet contained nuclei. The supernatant was clarified by centrifugation 18000 g, 5 min at 4°C to separate the mitochondria in the pellet from the cytosol and the cytoplasmic membrane in the supernatant. The cytosol and the cytoplasmic membrane were separated by ultracentrifugation 100000 g for 1 h at 4°C. The supernatant corresponding to the cytosol (Cs) was harvested and the pellet was washed once in the fractionation buffer before suspension in RIPA lysis buffer (Cp). The nuclei were washed as indicated in the first protocol on a cushion of fractionation buffer, suspended in RIPA lysis buffer and sonicated (N).

For protein dosage and loading, all the fractions were treated as indicated for whole cell lysates.

Proteins separated on 10% SDS-polyacrylamide gels were transferred on Hybond ECL membranes (GE Healthcare). Transfer efficiency of the proteins was checked by transient staining of the membrane using Ponceau's red (Sigma). Blots were submitted to Western analysis using the following antibodies: anti-HP1γ (clone 2MOD-1G6AS, Euromedex), anti-HA (HA.7, Sigma), anti-β tubulin (Sigma), anti-HSP90 (AC88, Enzo), anti-LaminB1 (Abcam), 2MeH3K4 (Upstate), 3807 anti-ENS-1 polyclonal antibody. Anti-mouse and anti-rabbit secondary antibodies coupled with peroxidase were from Sigma. Saturation of the membrane was performed in saturation buffer (0.1% Tween, 5% low fat milk in PBS) 1 h at room temperature. Antibodies were diluted in saturation buffer and incubated with the membrane at room temperature for 1 h. After washing 3 times for 10 min in PBS-0.1% Tween, the membrane was incubated 1 h at room temperature with the HRP-conjugated secondary antibodies diluted in saturation buffer. Anti-mouse and anti-rabbit HRP conjugates were from Promega. After washing, peroxydase activity was revealed using the Amersham ECL western blotting detection reagent (GE Healthcare) as substrate and X-ray films (Fuji).

### CAT assay

CES cells seeded in 12-well plates were co-transfected at 70% confluency using Lipofectamine 2000 (Invitrogen) as described by the manufacturer with a mixture of the following plasmids: pM plasmid (20 ng) encoding for proteins in fusion with Gal4 DNA binding domain (GBD-proteins), pG4-TK-CAT reporter plasmid (300 ng) allowing the tethering of the GBD-proteins upstream the TK promoter that controls the expression of the CAT gene, pCi plasmid (200 ng) allowing the expression of additional proteins and pRL-CMV plasmid (20 ng) as control to normalize transfection efficiency. After 24 h culture, the medium was removed and the cells were washed twice with PBS. Cell lysates were prepared and CAT expression was measured using the CAT ELISA kit from Roche according to the manufacturer's instructions. Renilla luciferase luminescence was measured on templates from the same lysates using the Renilla Luciferase Assay System (Promega) according to the manufacturer's protocol and using a Mithras LB940 microplate reader (Berthold technologies).

### In silico analysis of the Ens-1 gene copies in the chicken genome

We used the sequence of Soprano [19] as a query to search the chicken genome (version WASHUC 2, retrieved from Ensembl http://www.ensembl.org/index.html) using BLASTN [20]. We recovered 78 copies among which 25 were very conserved compared to the reference sequence. We used ORF Finder (http://www.ncbi.nlm.nih.gov/gorf/gorf.html) on the 25 conserved sequences to determine the presence of ORFs and to retrieve the subsequent protein sequences. The coiled coil domains were predicted on the protein sequences using the MultiCoil program [21].

### *In vitro* transcription

Capped RNAs were obtained by using 2 μg of linear DNA template mixed with 20 U of T7 RNA polymerase (Promega Co., Madison, WI, USA), 40 U of RNAsin (Promega Co, Madison, WI, USA), 10 mM rATP, rUTP, rCTP, 0.48 mM rGTP, 30 mM DTT in transcription buffer [40 mM Tris–HCl (pH 7.9), 6 mM MgCl2, 2 mM spermidine and 10 mM NaCl], the m7GpppG cap analogue (Invitrogen, Co) was added to a final concentration of 1.92 mM as previously described [22]. The transcription reaction was carried out at 37°C for 1 h and the RNAs were precipitated with LiCl at 2.5 M final concentration. The integrity of the RNAs was checked by electrophoresis on non-denaturating agarose gels and their concentration was quantified by spectrophotometry at 260 nm using Nanodrop (NanoDrop Technologies, Wilmington, Delaware, USA).

### *In vitro* translation

*In vitro* transcribed RNAs were translated in 10 μl of the supplemented untreated RRL 50% (v/v) (Promega Co., Madison, WI, USA) in the presence of KCl (75 mM), MgCl_2_ (0.5 mM), 20 μM of amino acids mix minus methionine and 0.6 μCi of [^35^S]-methionine (GE Healthcare Life Sciences) for 30 min at 30°C. Reactions were stopped with 2× SDS-loading buffer and the products were resolved by 15% SDS–PAGE. Gels were dried and subjected to autoradiography using Biomax films (Eastman Kodak Co.).

## Results

### ENS-1 can control HP1γ activity in ES cells

**Figure 1:**
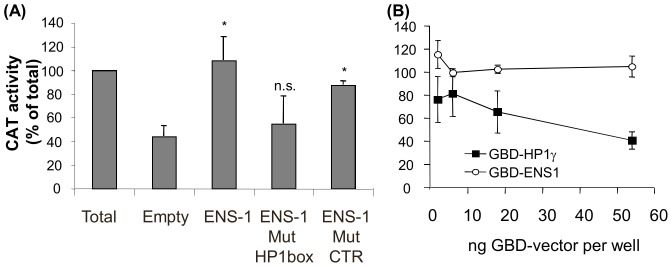
Transcriptional repression by HP1γ is modified by its interaction with ENS-1 in CES. CES cells were co-transfected with the pG4-TK-CAT reporter plasmid and the plasmid encoding for the indicated protein in fusion with a Gal4-DNA binding domain (GBD). An equal amount of pRL-CMV plasmid encoding for Renilla Luciferase was added to each well for normalization. (A) Transcription inhibition of the CAT reporter gene by GBD-HP1γ in the presence of pCi expression vectors encoding for ENS-1, for ENS-1 mutated in the HP1box. Mutation in an irrelevant PxVxL sequence was used as control. Maximal inhibition by HP1γ was given by co-transfection with the empty pCi vector (Empty). (B) Promoter activity was compared in the presence of GBD-ENS-1 and GBD-HP1γ. In (A) and (B) the promoter activity was assayed by quantifying the amount of CAT protein expressed by CAT-ELISA. Data were normalized on the basis of the luciferase activity in each well. Results represent CAT expressions in the presence of GBD-fusion proteins as percent of the maximal expression obtained with GBD alone in the same conditions. They are mean of four (A) and two (B) independent experiments +/− SD. Statistics are results of t test relative to the value obtained in the Empty condition: * p<0.005; n.s. not significant (p>0.1). In (B), CAT activity difference between 2 and 6 ng of GBD-ENS-1 vector is not significant.

The control exerted by ENS-1 on the transcriptional regulation mediated by HP1γ has been demonstrated in the neural plate [7]. Here we investigated whether this activity might also occur in chicken ES cells (CES). To this end we used a CAT reporter gene placed under the control of a minimal VP16 promoter downstream of the DNA motif recognized by the Gal4 protein. Cells were co-transfected with a pM vector encoding for HP1γ in fusion with the DNA binding domain of Gal4 (GBD) to target its interaction with the CAT reporter plasmid. In line with results from another study [23], Figure 1A shows that transcription was repressed when tethering HP1γ to the promoter. Transfection of CES cells with the previous cocktail supplemented with an ENS-1 expression vector restored the transcriptional activity to levels obtained in the absence of HP1γ while ENS-1 mutated in the HP1 box had no effect (Fig. 1A). To test whether ENS-1 acts directly on the promoter as a transcriptional activator, ENS-1 in fusion with the GBD was co-transfected with the CAT reporter plasmid. Transcription was not affected by the recruitment of ENS-1 to the promoter (Fig. 1B) confirming that the effect of ENS-1 on transcription was dependent on its interaction with HP1γ.

Therefore ENS-1 can directly interact with HP1γ and modulate its function as a transcriptional regulator in CES.

### ENS-1 is both a cytoplasmic and nuclear protein

**Figure 2:**
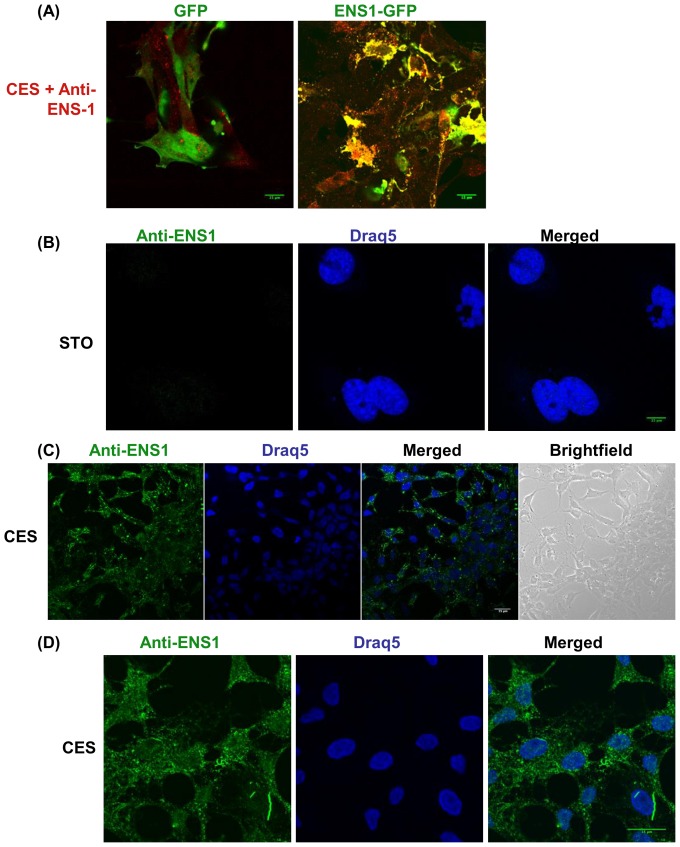
Localization of ENS-1 in the cytoplasm and in the nucleus of CES. (**A**) CES transfected with an expression vector for GFP alone or in fusion with the ENS1 protein were incubated with the 16h4antibody produced against ENS1. An anti-mouse antibody coupled with Alexa555 was used as secondary antibody (red) while GFP proteins were in green. Overlapping signals gave a yellow staining. In (**B**), (**C**) and (**D**) immunostaining with the 16h4 antibody was detected with an Alexa488 anti-mouse antibody (green) and the nucleus was localized by DNA staining with Draq5 (blue). (**B**) The 16h4 anti-ENS-1 antibody does not label STO murine embryonic fibroblasts (used as negative control). (**C**) ENS-1 detected in CES cells cultivated in proliferating conditions. (**D**) ENS-1 in CES cells at a higher magnification. All observations were performed by confocal microscopy. Is shown: the signal over the background obtained with the secondary antibodies in the absence of primary antibody. Scale bar 15 μm. The images are representative of three independent experiments.

Since HP1γ is a nuclear protein, we looked for the distribution of the endogenous protein ENS-1 in these cells. To this end a monoclonal antibody was produced against the recombinant protein ENS-1 (as described in Material and Methods) and tested for use in confocal microscopy. Among a dozen of clones reacting with the immunogen, only one was suitable for immunostaining. Specificity for ENS-1 was first demonstrated by transfection of CES with the ENS-1-GFP fusion protein. As shown in Figure 2A, the antibody co-localized with ENS-1-GFP but not with GFP alone used as control. Secondly, as Ens-1 is restricted to the galliform species [5], we used STO murine embryonic fibroblasts as negative control. No signal was observed with the antibody in these cells (Fig. 2B). Therefore the antibody is specific for ENS-1 and represents a relevant tool to study expression and distribution of this protein in CES. As shown in Figure 2C almost all the CES cells expressed ENS-1 but inside the cells the labeling was not uniform, notably in the cytoplasm when compared with the nucleus (see Fig. 2D). In the cytoplasm the distribution of ENS-1 appeared as organized, forming a scaffold of spots and stretches that sometimes crossed-over the nuclear membrane. In the nucleus that was labelled with Draq5, a stoechiometric DNA-intercaling agent, the distribution of ENS-1 was more uniform. Nucleolus that were not labeled with Draq5 were poorly stained with the anti-ENS-1 antibody (Fig. 2D).

**Figure 3:**
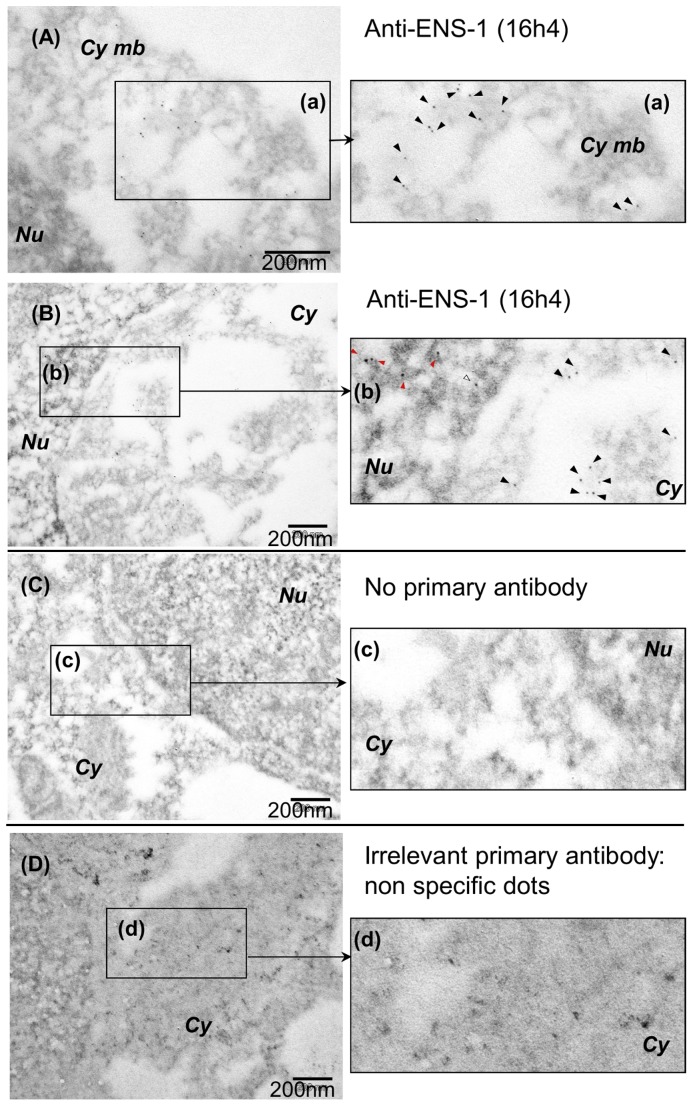
Subcellular localization of ENS-1 by electron microscopy. CES were labeled with the 16h4 anti-ENS1 antibody. The secondary antibody was a gold-conjugated anti-mouse antibody (gold particles are seen as black dots). To preserve the affinity of the antibody for ENS-1 a moderate fixative procedure (4% PFA) was performed. Some sub-cellular structures were not well preserved and showed an empty appearance in the cytoplasm. (**A**) Observation of ENS-1 close to the cytoplasmic membrane *Cy mb*. (a) Magnification of the rectangle designed in (A).Black arrowheads show the 16h4 dots. (**B**) Observation at the junction between the cytoplasm *Cy* and the nucleus *Nu.* (b) Magnification of the rectangle designed in (B) shows ENS-1 in the chromatin (red arrowheads), in interchromatin spaces (white arrowheads) and in the cytoplasm (black arrowheads). (C) No labeling was obtained in the absence of the 16h4 antibody nor (**D**) with the anti-human androgen receptor 441 used as negative control in the same conditions. (c) and (d) show magnification of the rectangles designed in (C) and (D) respectively. Non specific dots with the irrelevant primary antibody in (D) and (d) were larger and have irregular outlines when compared with the 16h4 antibody (C) and (c).

The distribution of ENS-1 was further examined by electron microscopy with the same antibody (Fig. 3). To preserve the intracellular structures this approach required fixative conditions that were stronger than those used for confocal analysis and thus altered the ENS-1 epitope recognized by the 16h4 antibody. A compromise was found using 4% PFA only. Most of the cell structure was preserved as well as the ENS-1 epitope but some cytoplasmic structures were lost, leaving empty areas in the cytoplasm that likely correspond to the endoplasmic reticulum (Fig. 3). ENS-1 was abundantly detected at the periphery of these areas (Fig. 3A) and in the nucleus (Fig. 3B). Examination of the cytoplasmic compartment shows that ENS-1 accumulates at the cytoplasmic membrane (Fig. 3A). In the nucleus, the protein was found in regions that were more or less dense to electrons, and corresponding to chromatin and to non-chromatin spaces respectively, suggesting that ENS-1 location in the nucleus is not restricted to protein complexes formed on DNA (Fig. 3B). No particles were observed in controls where anti-ENS-1 antibody was replaced by an irrelevant antibody (Fig. 3D) or in the absence of primary antibody (Fig. 3C).

Therefore, we conclude that the protein ENS-1 is expressed in all CES cells and is largely present in the cytoplasm and in the nucleus.

### ENS-1 sparsely co-localizes with HP1γ in the nucleus and in the cytoplasm

**Figure 4:**
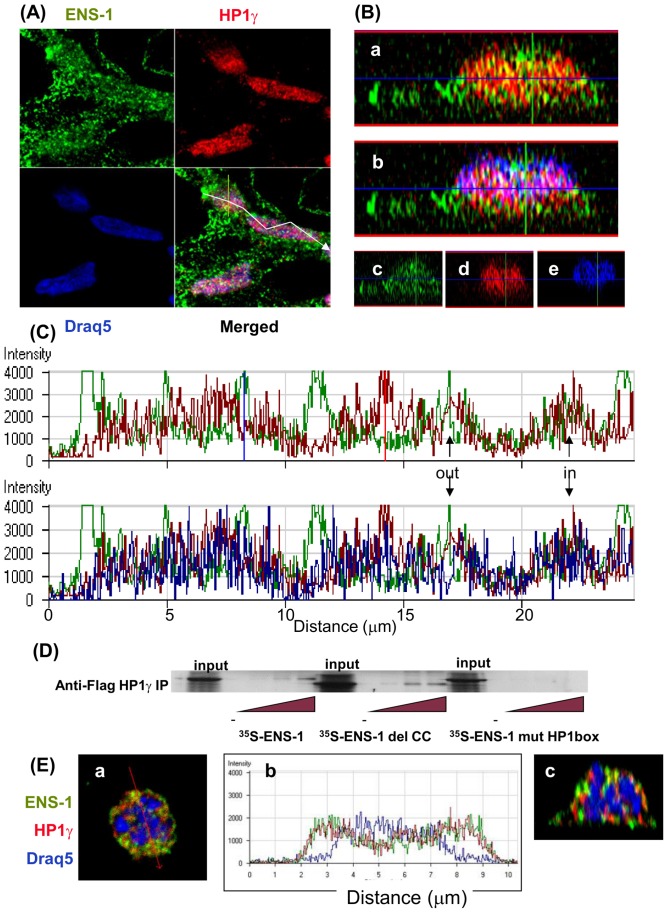
Sparse co-localization of ENS-1 and HP1γ in CES nuclei. (**A**) Immunostaining of CES cells was performed simultaneously with the rabbit anti- HP1γ (red) and the mouse anti-ENS-1 antibodies (green) detected using Alexa 555 and Alexa 488 conjugated secondary antibodies respectively. DNA was stained with Draq5 (blue). Observations were performed by confocal microscopy. (**B**) Distribution of ENS-1 and HP1γ in the thickness of CES. The z-cut axis is reported in (A) with a yellow bar on the merge panel. Distribution of HP1γ and ENS-1 is shown in panel a, co-localization of both proteins gives a yellow spot. In panel b the DNA staining was shown and white spots indicate ENS-1 and HP1γ proteins co-localizing on DNA. Individual distributions of ENS-1, HP1γ and DNA merged in a and b are represented in panels c, d and e respectively. (**C**) Fluorescence intensity for the three labels along the line represented in (A) by the white arrow. The upper panel represents fluorescence intensities obtained for HP1γ (red) and ENS-1 (green), the intensities obtained for chromatin (blue) have been added to the lower panel. Arrows point out co-localizations of HP1γ and ENS-1 in or out the chromatin. Intensity axis is in arbitrary units. (**D**) Pull-down experiments of ^35^S-ENS-1 with Flag-HP1γ. Lysates from COS cells transfected with pCi-flag- HP1γ were used as a source for the flag tagged chicken HP1γ protein. ^35^S labeled ENS-1 proteins were obtained by *in vitro* translation from pGBKT7 constructs coding for intact or mutated ENS-1 proteins: del CC indicates deletion of the coiled-coil domain (-50AA, 5.56 kDa), mutHP1 box indicates mutation in the HP1 box. After incubation of COS7 cell lysates with different dilutions of the ENS-1 proteins, HP1γ was precipitated with an anti-flag antibody and the association with ^35^S-ENS-1 was revealed by fluorography. Representative results of at least three different experiments. (**E**) Confocal analysis of a CES cell entering in mitosis (prometaphase) from the same experiment as in A. In panel a, merge representation of the labeling for ENS-1, HP1γ and DNA as in A. In panel (b), fluorescence intensity along the line represented in panel (a) by the red arrow for the three markers labeled. The panel (c) is a z-cut representation of the same cell.

To explore the context in which the interaction between the endogenous proteins ENS-1 and HP1γ may occur, co-localization experiments were performed in CES. Consistent with a previous study in mouse ES cells [24], immunostaining of HP1γ heterogeneously formed a speckled and diffuse pattern in the nucleus of CES (Fig. 4A). Yellow spots in the orthogonal sections of the cells (Fig. 4B, panel a) indicated that both proteins co-localized in interphasic nuclei while other ENS-1 and HP1γ molecules were visualized as neighboring green and red spots respectively. Merging with the DNA staining, in blue, produced white spots reflecting co-localizations of ENS-1 with HP1γ in chromatin (Fig. 4B, panel b) and a majority of purple spots corresponding to HP1γ on chromatin. These results are in agreement with the ability of ENS-1/HP1γ heterodimers in the embryo to specifically regulate gene transcription [7]. Panels c, d and e, showing each of the labeling that were merged in panels a and b (Fig. 4B), revealed that ENS-1 was present in all the thickness of the nucleus (panel c) while HP1γ and the chromatin had a more restricted distribution. Notably HP1γ proteins were concentrated in the center of the cells (panel d) while chromatin was mostly observed in the upper half (panel e). Measurement of the fluorescence intensity along the longitudinal axis of two cells confirmed that most of the HP1γ dots did not overlap with those of ENS-1 (Fig. 4C, upper panel) and that co-localization between both proteins was a restricted event that also occurred in regions with low DNA density (Fig. 4C, lower panel). These results suggest that ENS-1/HP1γ heterodimers can exist independently on the interaction with chromatin. In agreement with this hypothesis, pull-down experiments performed between in vitro translated ^35^S-ENS-1 and cell lysates from COS7 cells overexpressing chicken HP1γ demonstrate that the interaction between both proteins is direct, involving the HP1 box of the ENS-1 protein but not its coiled-coil domain as illustrated by the use of mutated ENS-1 proteins (Fig.4D). During mitosis, HP1γ is released from the chromatin and is distributed diffusely throughout the cell [25]. Accordingly, in prometaphasic cells when the nuclear membrane was dissolved, HPγ and ENS-1 were progressively released from the condensing chromatin and accumulated in the cytoplasm (Fig. 4E). Co-localizations were observed (Fig. 4E panel b) but most of the proteins were maintained separated as illustrated by the few number of yellow spots in comparison with the red and green ones (see panels a and c, Fig. 4E). In interphasic cells some HP1γ molecules were also detected in the cytoplasm as confirmed by the use of two distinct antibodies (Fig. S1) and a partial co-localization with ENS-1 was found in this compartment (Fig. S2 a).

Therefore the interaction between ENS-1 and HP1γ does not require any CES specific co-factor nor chromatin binding and might occur in the cytoplasm and in the nucleus. However despite a close contact between both proteins outside the nucleus during mitosis, the majority of them co-localize in the nucleus of interphasic cells.

### ENS-1 protein is differently regulated in the cytoplasm and in the nucleus

**Figure 5:**
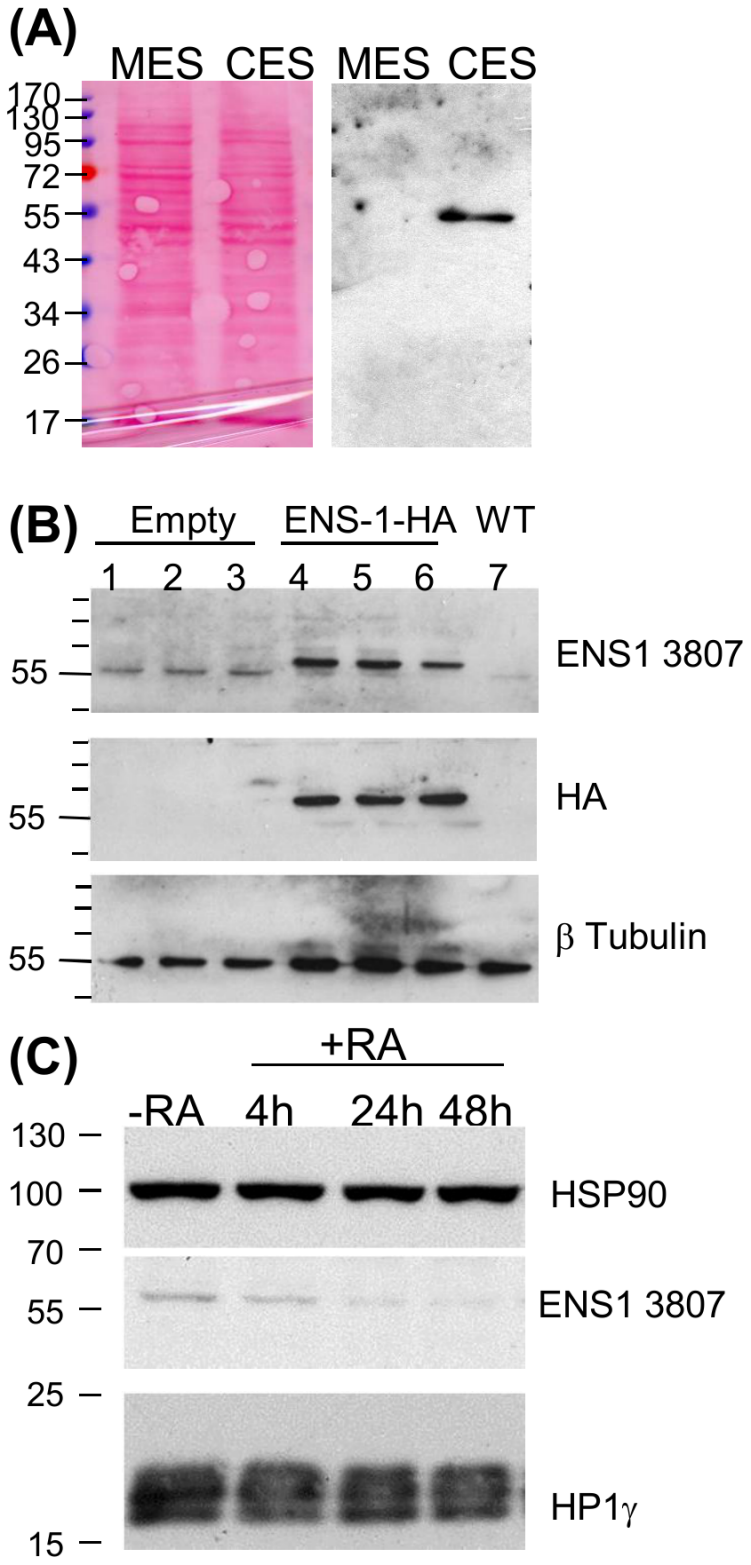
Western blot analysis of the protein ENS-1. (**A**) Proteins (15 μg) of whole cell lysates from murine (MES) and chicken (CES) cells were analyzed by western blot using the anti-ENS-1 3807 antibody. Protein loading was equivalent in both conditions as illustrated by the Ponceau's red staining of the blot. (**B**) Proteins lysates (20 μg) from CES cells transfected with an HA-tagged ENS-1 protein (lanes 4,5,6) or with an empty vector (lanes 1,2,3) were compared with untransfected cells (WT, lane 7). Triplicates were from three independent transfection experiments. The HA-tagged protein was detected by the 3807 antibody and by the anti-HA antibody at a molecular weight of 60 kDa. The anti-ENS1 antibody also detected the endogenous protein at 55 kDa. Both proteins differed in size by 3 kDa corresponding to the 33 additional amino acids added at the C-terminal part of ENS-1 in the transgenic protein in addition to the two HA tags (2.4 kDa). (**C**) Western blot analysis of ENS-1 and HP1γ (Chemicon antibody) in whole cell lysates (12 μg) from undifferentiated CES (-RA) or CES induced to differentiate with retinoic acid (RA, 10^−6^M) for 4 to 48 h as indicated. HSP90 was used as protein loading control.

To better characterize the protein ENS-1 in the cytoplasm and in the nucleus, we performed western blot analysis of whole cell lysates. To this end we developed another ENS-1 specific antibody called 3807 (see Material and Methods). As shown in Figure 5A, this antibody gave a unique band of about 55 kDa in CES, but not in mouse ES cells, indicating its specificity for chicken cells. This molecular weight is in accordance with the 54 kDa prediction made from the protein sequence (490aa, Genebank: AAK06824). To confirm that the protein recognized by the 3807 antibody is ENS-1, CES cells were transfected with an expression vector (pCx ENS-1-HA-ires-puro) encoding for an HA-tagged ENS-1 protein. To be sure that antibiotic resistant cells will also express Ens-1 transcripts, both genes were placed under the control of the same promoter and were separated by an IRES sequence to generate a unique transcript for both genes that are next translated independently. Indeed, culture with puromycin of CES cells transfected with pCx ENS-1 but not with the empty vector, induced the expression of a unique HA-tagged protein as observed with the anti-HA antibody in Figure 5B. This protein is also recognized by the 3807 antibody in addition to the endogenous ENS-1 protein detected below and in control cells. These results confirm the specificity of the 3807 antibody for ENS-1.

**Figure 6:**
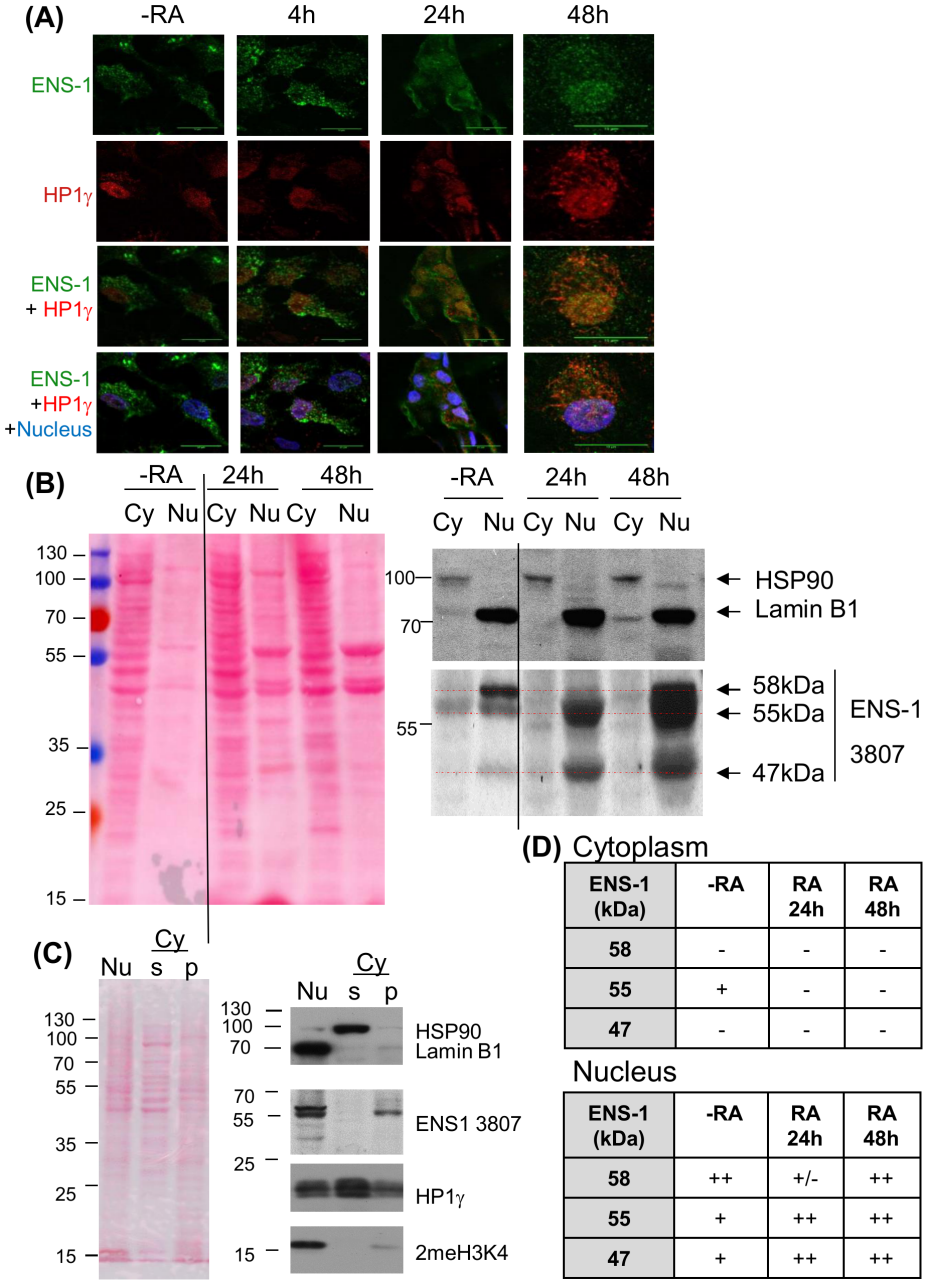
Subcellular distribution of ENS-1 in CES and during differentiation. (**A**) Immunostaining of ENS-1 (16h4 antibody) and HP1γ (Abcam antibody) in CES (-RA) and CES differentiated with retinoic acid for the indicated period. Nuclei are labeled with Draq5 and are in blue in merging panels. Image acquisition was optimized to observe the distribution of the proteins and does not reflect the real expression level. Scale bar 15 μm. (**B**) Western blot analysis of ENS-1 in the cytoplasmic (Cy) and in the nuclear (Nu) fractions of undifferentiated CES (-RA) or CES induced to differentiate with retinoic acid for 24h or 48h. Ponceau's red staining serves as a protein loading control between both fractions. In the nucleus soluble and insoluble components were separated and only the precipitating fraction that contains ENS-1 is shown. The volume corresponding to 6×10^6^ cells was loaded for the N fraction and Lamin B1 was used as loading control. In the cytoplasm (Cy) ENS-1 was in the supernatant, 15 μg of proteins were loaded and HSP90 was used as loading control. The vertical line indicates missing lanes between the two presented parts of the same gel. Dotted lines in red indicate the position of three ENS-1 related proteins. (**C**) Separation of soluble and insoluble components from the cytoplasm. The cytoplasmic fraction separated from the nucleus was subjected to extended centrifugation. The pellet (Cy p) and the supernatant (Cy s) were analyzed for ENS-1 protein and HP1γ. The nuclear fraction corresponding to the whole nuclear proteins is represented (Nu). Protein loading was 10 μg for each fraction. HSP90 identifies the cytoplasmic soluble fraction, Lamin B1 identifies fractions containing membrane proteins and 2MeH3K4 identifies fractions containing chromatin. Results in (B) and (C) were obtained from two independent experiments using distinct fractionation protocols. (D) Summary tables of the ENS-1 forms found in the cytoplasm (upper table) and in the nucleus (lower table) from CES cells (-RA) and from CES induced to differentiate with retinoic for 24 h or 48 h.

In a previous report we have shown that the promoter of *Ens-1* is repressed while the expression of Sox2 is induced when CES differentiate [8]. These results are in agreement with the idea that differentiation of CES *in vitro* may mimic the release of the repression mediated by the dimer ENS-1/HP1γ on the promoter of Sox2 before the emergence of the neural plate. To explore the relevance of this hypothesis, variations of ENS-1 expression during differentiation were examined at the protein level. Western-blot results in Figure 5C show that the protein content decreased to become quite undetectable 24 h after retinoic acid addition and was maintained at this residual level after 48 h. In the same samples the expression level of HP1γ was not affected by differentiation (Fig. 5C) in agreement with previous observations in mouse ES cells [12]. These results are in accordance with the known regulation of the promoter of Ens-1 [8]. To confirm with another approach, ENS-1 expression was analyzed during CES cells differentiation by immunofluorescence experiments using the 16h4 antibody. This approach confirmed the decrease of the cytoplasmic ENS-1 during differentiation but with changes in the distribution of ENS-1 (Fig. 6A). In contrast to undifferentiated cells where the protein was mostly cytoplasmic, the localization was mostly nuclear in differentiated cells (Fig. 6A, Fig. S2). Notably, the organized distribution of ENS-1 in the cytoplasm was lost, giving a diffuse signal after 24 h differentiation that became undistinguishable from the back-ground after 48 h while the signal in the nucleus was still over the background (Fig. 6A). These results suggest that variations in ENS-1 contents are not similar in the cytoplasm and in the nucleus.

To further explore these changes, enrichments in the nuclear and in the cytoplasmic proteins were obtained by fractionation of cell lysates before analysis by western blot using the 3807 antibody.

Results in Figure 6B show that the 55 kDa ENS-1 protein is present in the cytoplasm and in the nucleus of undifferentiated cells. However in the nucleus only, this band is flanked by two other ones of 58 and 47 kDa that were not detected when using whole cell lysates. During differentiation, the 55 kDa protein was decreased in the cytoplasm but maintained in the nucleus. This was also generally true of the two other proteins. More precisely, the relative proportions of the three ENS-1 related bands in the nucleus were modified during differentiation. In undifferentiated cells, the 58 kDa nuclear protein was more abundant than the two others. After 24 h differentiation, the proportion of the 55 and the 47 kDa bands increased relatively to the 58 kDa band and became more abundant. Later, in 48h-differentiated cells, all three proteins were equally and strongly detected in the nucleus (Fig. 6B). Altogether these results confirm the observations made by immunostaining with the 16h4 antibody and indicate that the 55 kDa protein ENS-1 is maintained in the nucleus but not in the cytoplasm upon differentiation. They also reveal that ENS-1-like proteins recognized by the 3807 antibody are expressed and are restricted to the nucleus.

It is of note that all these proteins are associated with the insoluble components of the nucleus (Fig. 6B) and were not found in the soluble fraction (Fig. S3). The decrease observed in Figure 5C with whole cell lysates reflects the cytoplasmic ENS-1 protein only since insoluble components were discarded by centrifugation before analysis.

To confirm that the cytoplasm does not contain the additional 47 and 58 kDa proteins detected in the nucleus, a fractionation protocol disrupting the cells without detergent was managed to preserve the soluble and the insoluble parts of the cytoplasm that were next separated by centrifugation. Results in Figure 6C show that ENS-1 was restricted to the pellet confirming that ENS-1 is not a soluble protein. Contamination with nuclear components was excluded since a unique band of 55 kDa was recognized with the anti-ENS-1 antibody and only traces of histone (2MeH3K4) and laminB1 coming from the nucleus were found. In parallel, analysis of the nuclear content showed the additional bands already observed with the previous protocol. The homogenous protein loading rendered possible the quantitative comparisons between the distinct sub-cellular fractions. The 55 kDa protein ENS-1 was equivalently distributed between the cytoplasm and the nucleus in CES cells. In the nucleus only, the 47 kDa was present but poorly detectable and the abundance of the 58 kDa was confirmed. Interestingly, HP1γ was found in all fractions, in agreement with immunofluorescence data. Therefore, it seems that differently from HP1γ, ENS-1 is not a free protein in the cytoplasm nor in the nucleus but is rather associated with insoluble structures.

Altogether the results obtained by a combination of immunofluorescence and western blot approaches using two distinct ENS-1 specific antibodies support the conclusion that the endogenous protein ENS-1 is differently regulated in the cytoplasm and in the nucleus and that ENS-1-like proteins also contribute to this difference between both compartments.

### In silico analysis of Ens-1 gene copies

Due to its retroviral origin, distinct copies of the gene Ens-1 exist [5] that may generate distinct ENS-1 like proteins depending on the conservation of their ORF sequence. Those with the most conserved ORF in an updated sequence of the chicken genome are listed in Table 1. Seven copies encode for a 54 kDa ENS-1 protein and one copy for an ENS-1 like protein of 47 kDa but none for a 58 kDa protein. Based on a full sequence homology with the 3807 peptide and on conservation of the promoter active domains [8], the 47 kDa and five of the 54 kDa proteins encoding copies may account for the protein pattern detected with the 3807 antibody. Copies encoding for lower or for higher molecular weight proteins ranking from 34 to 69 kDa fulfill the same criteria but were not detected by this antibody (see Fig.5A, 6C and S3 to compare distinct cell lysis protocols). Of note, all the ENS-1 like proteins with an intact 3807 sequence have a conserved coiled-coil domain but only the 54 kDa proteins have an intact HP1box. Thus, it can be assumed that ENS-1-like proteins would be inactive towards HP1γ if generated from distinct gene copies.

**Table 1:** List of the ENS-1 like sequences and homology with the 3807 peptide.

Sequence	Position of the gene			Protein Size		AA	Promoter
Name	Chrom.	Start	End	Amino Acids (AA)	kDa (1)	Identity (2)	activity (3)
Seq.12	chr1	153080201	153086086	490	54	16/16	Y
Seq.7	chr1	105099609	105104886	490	54	16/16	Y
Seq.32	chr2	95636316	95642444	488	54	16/16	Y
Seq.62	chrUn_ran.	23794802	23799438	487	54	16/16	Y
Seq.10	chr1	146896807	146900813	490	54	16/16	No
Seq.31	chr2	95607871	95610941	427	47	16/16	Y
Seq.38	chr4	29062470	29069620	392	43	16/16	Y
Seq.41	chr5	56151532	56156408	475	52	16/16	Y
Seq.46	chr9	3145510	3148390	627	69	16/16	Y
Seq.52	chrUn_ran.	14717478	14720393	307	34	16/16	Y
Seq.30 (ENS-3)	chr2	72991670	73001955	698	77	16/16	Y
Seq.18	chr1	166123942	166128607	490	54	15/16	Y
Seq.2	chr1	49392030	49395290	490	54	15/16	No
Seq.69	chrUn_ran.	48116551	48120222	265	29	15/16	Y
Seq.54	chrUn_ran.	18990010	18992568	160	18	15/16	Y
Seq.15	chr1	164856462	164859682	213	23	N/A	Y
Seq.34	chr2	138621760	138623256	210	23	N/A	Y
Seq.26	chr2	36219087	36223257	378	42	N/A	Y
Seq.24	chr2	11703874	11708540	293	32	N/A	Y
Seq.40	chr5	3991240	3997526	182	20	N/A	Y
Seq.8	chr1	134745214	134749487	182	20	N/A	Y
Seq.22	chr12	18117921	18121939	182	20	N/A	Y
Seq.35	chr3	92605110	92611133	182	20	N/A	Y
Seq.20	chr1	167194063	167197535	182	20	N/A	No
Seq.3	chr1	49395084	49396779	<10	N/A	N/A	Y

Among 78 copies detected in the chicken genome, 25 were very conserved compared to the reference sequence and are listed below. ORF Finder was used to determine the presence of ORF and to retrieve the subsequent protein sequence. Identity with the 3807 peptide (16 amino acids) defined three categories: total identity (16/16 AA), partial identity (15/16 AA) or no identity (N/A).

(1) The molecular weight of the protein was calculated with the formula: AA number X 110/1000.

In bold are indicated the protein sizes detected in CES cell lysates with the 3807 antibody in western-blotting.

(2) Identity between the ENS-1 like protein sequences and the 3807 peptide (DRIRVLQNEARTRAGK)

(3) Potential activity based on the presence (Y) or the absence (No) of the Nanog, Gata and Ets transcription factors binding sites controling promoter activity.

**Figure 7:**
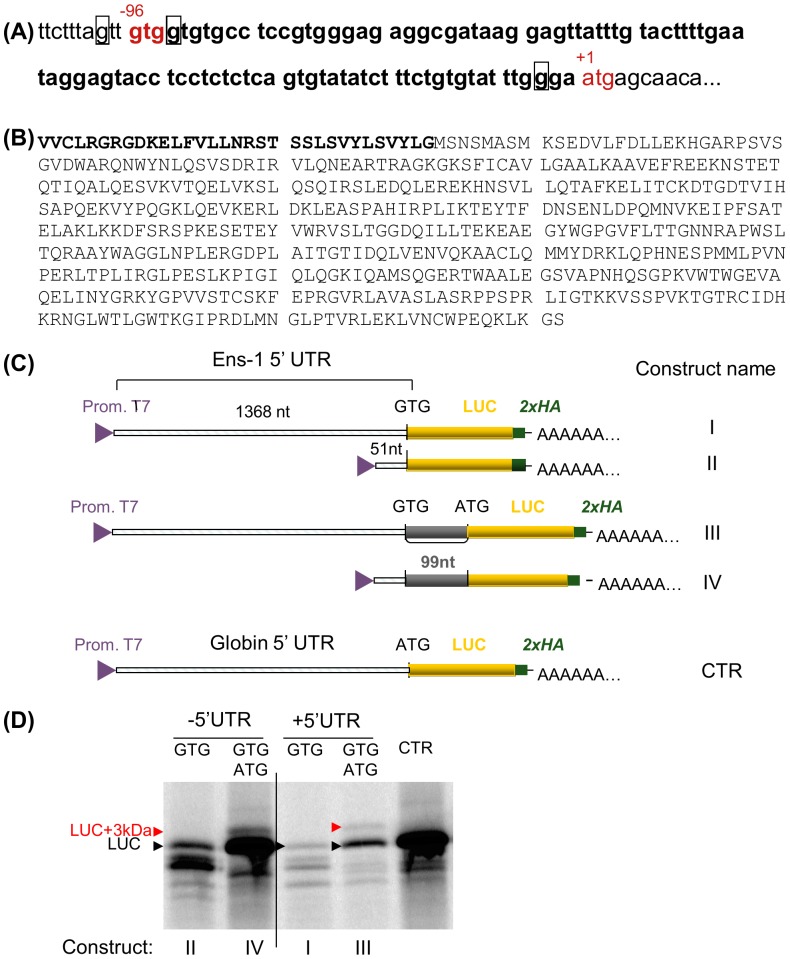
Presence of two start codons in the Ens-1 5′UTR. (**A**) The region containing the two putative start codons in the sequence encoding the whole mRNA (NM_001080873) is represented. The ATG start codon (in red) is the authentic initiation site and generates the 54 kDa protein that has been published previously (NM_001080873.1). The GTG codon (in red) is positioned 96 nucleotides. upstream of the AUG and generates a 57 kDa protein. The purine in positions -3 or +4 from the first base of each start codon is squared (Kozak consensus sequence). (**B**) Putative sequence of both proteins that only differ by the 3 kDa peptide in N-terminal position represented in bold. (**C**) Schematic representation of the constructs used to validate the translation initiation from the GTG and the ATG start codons in the 5′ UTR of Ens-1. (**D**) RNA generated from the constructs depicted in (C) were used for in vitro translation of the luciferase protein in the presence of radioactive methionine. [^35^S]methionine-labelled proteins were separated on SDS-PAGE and revealed by autoradiography. The position of the luciferase protein (LUC) generated by constructs with only one start codon is indicated (GTG in Ens-1 5′UTR or ATG in CTR). Initiation from the two start codons generates LUC with ATG and a larger protein (LUC+3 kDa) resulting from initiation at the upstream GTG.

Alternatively, the 58 kDa ENS-1 like protein may result from translation initiation starting from a start codon distinct from the ATG that accounts for the 54 kDa protein. This hypothesis is supported by the existence of a GTG sequence in the 5′UTR of the gene Ens-1 that is located 96 nucleotides upstream the ATG (Fig. 7A) and may extend the Ens-1 coding sequence, as already reported for human genes [26]. Both codons are in frame and the N-terminal extension generated from the GUG would overweight the ENS-1 protein of 3 kDa (Fig. 7B), corresponding to the difference between the 55 and the 58 kDa proteins detected by western blot. We checked the translation efficiency from the GUG codon alone or in the presence of the ATG. Using the constructs containing the 5′UTR of ENS-1 upstream of a luciferase reporter gene under the control of a T7 promoter (Fig. 7C), we generated RNAs that were used for *in vitro* translation. Results in Figure 7D show that the GUG start codon efficiently initiated the translation of the luciferase gene (as indicated by the arrow (LUC) in lanes II and I). When the ATG start codon was also present, translation was initiated from both codons to yield two proteins that differ in size by 3 kDa. Luciferase was produced from the ATG while the upstream GTG was responsible for the synthesis of the extended protein by translation of the 99 nucleotides segment from GTG to ATG (in lanes IV and III). It is noteworthy that protein expression from the GTG was less efficient when the latter was placed upstream of the ATG. Interestingly, when the Ens-1 5′UTR was driving protein synthesis (constructs I and III), expression was severely reduced from both start codons and translation from GUG became residual. These data indicate that an N-terminal extension of ENS-1 during RNA translation may account for the 58 kDa ENS-1 like protein but the low activity of the GUG start in the context of the Ens-1 5′UTR suggests that an additional regulation of translation would be involved in CES for a real contribution to the protein pattern revealed with the 3807 antibody.

Finally, it cannot be ruled out that ENS-1 like proteins result from post-translational modifications of the ENS-1 protein itself. In yeast two-hybrid assay ENS-1 interacts with the conjugating enzyme Ubc9 (unpublished data) that transfers a SUMO protein to its protein substrate [27]. SUMO are about 10 kDa proteins in size, that is compatible with a modification of the 47 kDa ENS1-like protein. We found in the sequence of ENS-1 two sumoylation motifs that are conserved in all the copies with an intact 3807 peptide sequence (Table 2), thus supporting the potential involvement of post-translational modifications in the production of the 5 kDa protein. It is of note that one of the SUMO binding sites is located inside the coiled-coil domain involved in dimerization of the protein and in its recruitment to Sox2 promoter [7]. In addition, as SUMOylation has been reported to regulate intracellular distribution of proteins [28], this may explain the nuclear localization of ENS-1 in spite of lack of nuclear localization signal.

**Table 2:** Conservation of functional domains in ENS-1 like copies.

	Protein size	Domains conservation and their position (AA)			
	kDa	HP1box (1)	cc-domain (2)	Sumo site 1 (3)	Sumo site 2 (4)
Sop.12	**54**	469	92-126	180	96
Sop.7	**54**	469	92-126	180	96
Sop.32	**54**	467	92-124	178	96
Sop.62	**54**	466	92-123	177	93
Sop.10	54	469	92-126	180	96
Sop.31	**47**	/	109-144	198	114
Sop.38	**43**	/	96-123	180	96
Sop.41	**52**	/	92-126	180	96
Sop.46	**69**	/	92-126	180	96
Sop.52	**34**	/	95-123	180	96
Sop.30 ( = ENS-3)	**77**	/	422-450	507	423
Sop.18	54	469	92-126	180	96
Sop.2	54	469	92-126	180	96
Sop.69	29	/	92-124	180	96
Sop.54	18	/	85-124	/	92
Sop.15	23	/	/	36	/
Sop.34	23	/	/	77	/
Sop.26	42	357	/	68	/
Sop.24	32	272	/	/	/
Sop.40	20	161	/	/	/
Sop.8	20	161	/	/	/
Sop.22	20	161	/	/	/
Sop.35	20	161	/	/	/
Sop.20	20	161	/	/	/
Sop.3	N/A	113	/	/	/

In red are indicated the copies with an intact 3807 sequence and with an intact promoter.

(1) Based on the HP1 box motif involved in the interaction of ENS-1 with HP1γ: GLPTVRLE.

(2) Coiled-coil(cc) domain defined using the MultiCoil program.

(3) Sequence motif PLIKTEY.

(4) Sequence motif ESVKVTQ, inside the coiled-coil domain.

## Discussion

This paper describes the endogenous form of the protein ENS-1 that controls the timing of the neural plate emergence in chicken by interacting with the epigenetic regulator HP1γ. Using different approaches, based on new antibodies raised against ENS-1, we show that this protein is strongly expressed in pluripotent CES cells isolated from the epiblast, in agreement with its transcriptional pattern of expression [8]. These antibodies are specific for ENS-1 since they react with a tagged-ENS-1 transgene but not with cells devoid of the Ens-1 gene. Each antibody was screened for immunofluorescence and for western-blot applications but none worked in both approaches. Therefore both antibodies were used in parallel in western blot or in immunofluorescence to characterize the protein ENS-1. They gave converging results about localization of the protein both in the nucleus where HP1γ is concentrated and in the cytoplasm, but with distinct features as revealed by western blot. In the nucleus, but not in the cytoplasm, three anti-ENS-1 reactive bands were found. Our results support the idea that the relative proportion of these ENS-1 like proteins is part of the regulation of ENS-1.

Indeed, differentiation of ES cells using retinoic acid promotes their commitment preferentially toward the neurectoderm [12]. Under these conditions, the expression of ENS-1 at the transcriptional level is only partially repressed [8]. With both antibodies we show in parallel, that the ENS-1 protein content is decreased in the cytoplasm but not in the nucleus where it is maintained but with deep changes in the relative proportions of all three proteins.

In the prospective neural plate the recruitment of ENS-1 to the promoter of Sox2 represses the gene, and activation [29] [30] is induced by competition with the neo-synthesized protein BERT that releases ENS-1 [7]. In mice, Sox2 [31] is known to be, along with Oct4 [32] and Nanog [33], one of the key players of pluripotency that are repressed during differentiation. Differently, in CES cells we reported that Sox2 is induced upon differentiation [8], suggesting that ENS-1 is fully active to repress Sox2 in CES. In agreement, we show here that ENS-1 can control the function of HP1γ in a reporter system and that both proteins locally co-localize on chromatin in the nucleus of CES cells.

Identification of genes targeted by the ENS-1/HP1γ heterodimer in CES represents a future challenge to understand integration of these proteins in the pluripotency network of CES cells, and the data presented here strongly support the development of approaches directly addressing this question at the protein level. The development of additional antibodies suitable for immune-precipitation will be required, this cannot be achieved with the antibodies presented here. Targeting other epitopes of the protein for antibody preparation will also be essential to better characterize the nature of the ENS-1 like proteins. The analysis of the different Ens-1 gene sequences suggests that distinct copies of the gene Ens-1 or another translation initiation start could at least partially explain the ENS-1 protein pattern observed, but the contribution of post-translational modifications cannot be excluded.

In conclusion our results demonstrate that understanding the regulation of the protein ENS-1 is more complex than could be anticipated from analysis of its transcripts and this study provides new tools to track this important protein during chick development.

## Supporting Information

Figure S1 **Immunostaining of the chicken HP1γ with the commercial antibody.** (A) CES cells transiently transfected with HP1γ in fusion with GFP (green) were labeled with the Ab1080 (Abcam) antibody used in Fig. 4. (B) CES cells were labeled with a mixture of the rabbit Ab1080 (red) and the mouse 42s2 (green, Upstate) anti- HP1γ antibody. The secondary antibodies used were an anti-rabbit conjugated with Alexa 555 (red) and an anti-mouse antibody conjugated with Alexa 488 (green). Overlapping signals gave a yellow color. Results from two independent experiments are presented. In the nucleus both antibodies gave similar staining and both detected traces of HP1γ in the cytoplasm but with more or less intensity depending on the experiment.
(TIF)

Figure S2 **Distribution and colocalization of ENS-1 with HP1γ during differentiation of CES cells.** Images are those presented in Figure 5C complemented with fluorescence intensity for the three labeling along the line represented in the merged image by the red arrow. CES cells (a) or cells differentiated for 4 h (b), 24 h (b) or 48 h (d) with retinoic acid were stained as in Figure 4 with anti-ENS-1 (green) and anti-HP1γ antibody (red). Signal intensities are presented as arbitrary units.
(TIF)

Figure S3 **Western blot of soluble and insoluble protein fractions in the nucleus of CES cells.** The whole blot corresponding to Fig. 5D is represented. N1 to N3 and S1 to S3 are loading replicates of respectively the insoluble and the soluble fractions of the nucleus. The S fractions had proteins concentrations lower (5 μg) than the N fractions (15 μg) but even in the S3 fraction that was slightly colored by Ponceau's red, no ENS-1 protein was detected.
(TIF)
