# Supplementary figures and images for: Subcellular Localization of ENS-1/ERNI in Chick Embryonic Stem Cells
Source: PLoS One. 2014 Mar 18;9(3):e92039. doi: 10.1371/journal.pone.0092039 (PMC3958431; doi:10.1371/journal.pone.0092039)

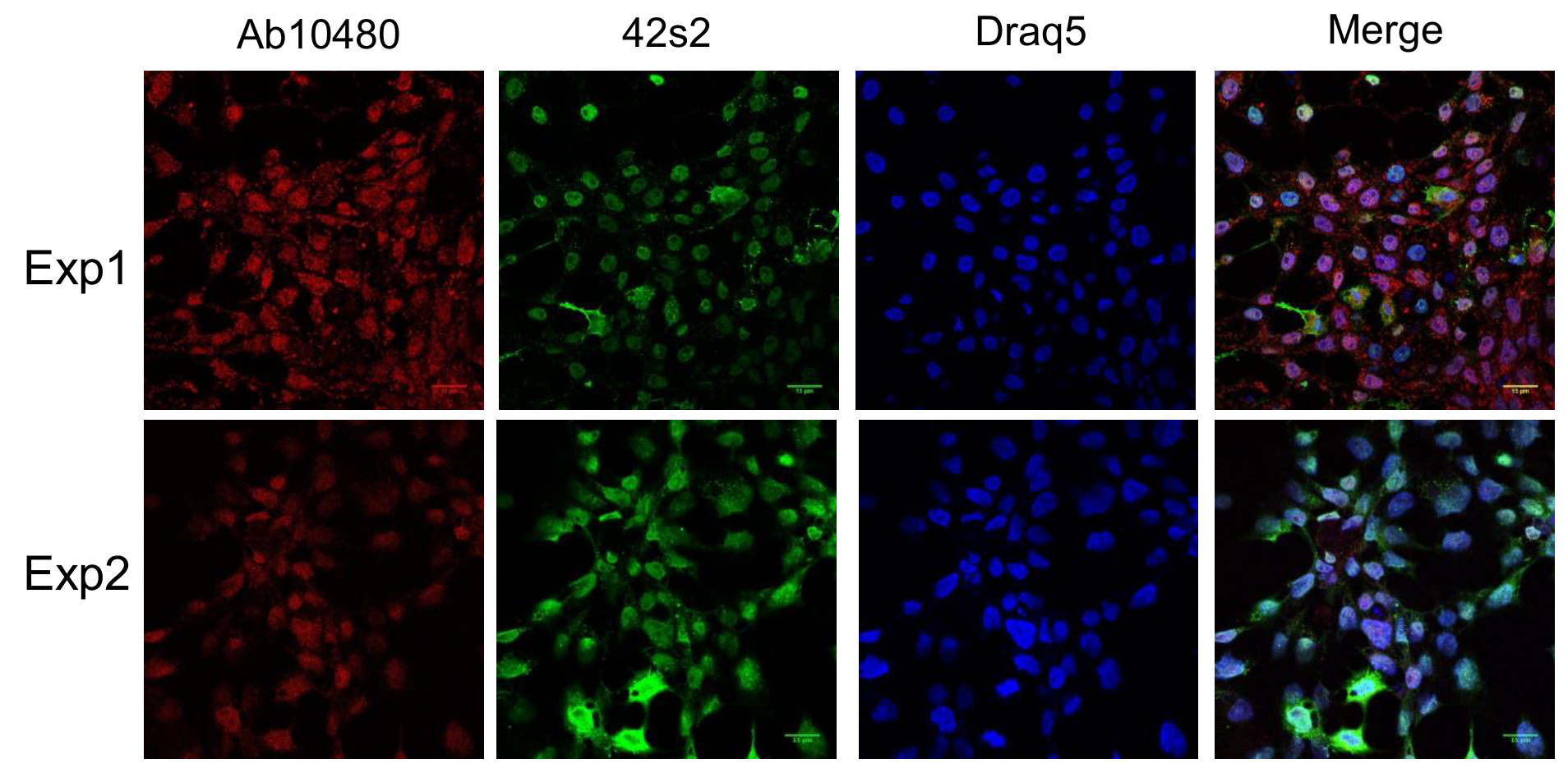

Supplement: Figure S1 — Immunostaining of the chicken HP1γ with the commercial antibody. (A) CES cells transiently transfected with HP1γ in fusion with GFP (green) were labeled with the Ab1080 (Abcam) antibody used in Fig. 4. (B) CES cells were labeled with a mixture of the rabbit Ab1080 (red) and the mouse 42s2 (green, Upstate) anti- HP1γ antibody. The secondary antibodies used were an anti-rabbit conjugated with Alexa 555 (red) and an anti-mouse antibody conjugated with Alexa 488 (green). Overlapping signals gave a yellow color. Results from two independent experiments are presented. In the nucleus both antibodies gave similar staining and both detected traces of HP1γ in the cytoplasm but with more or less intensity depending on the experiment. (TIF) [file pone.0092039.s001.tif]

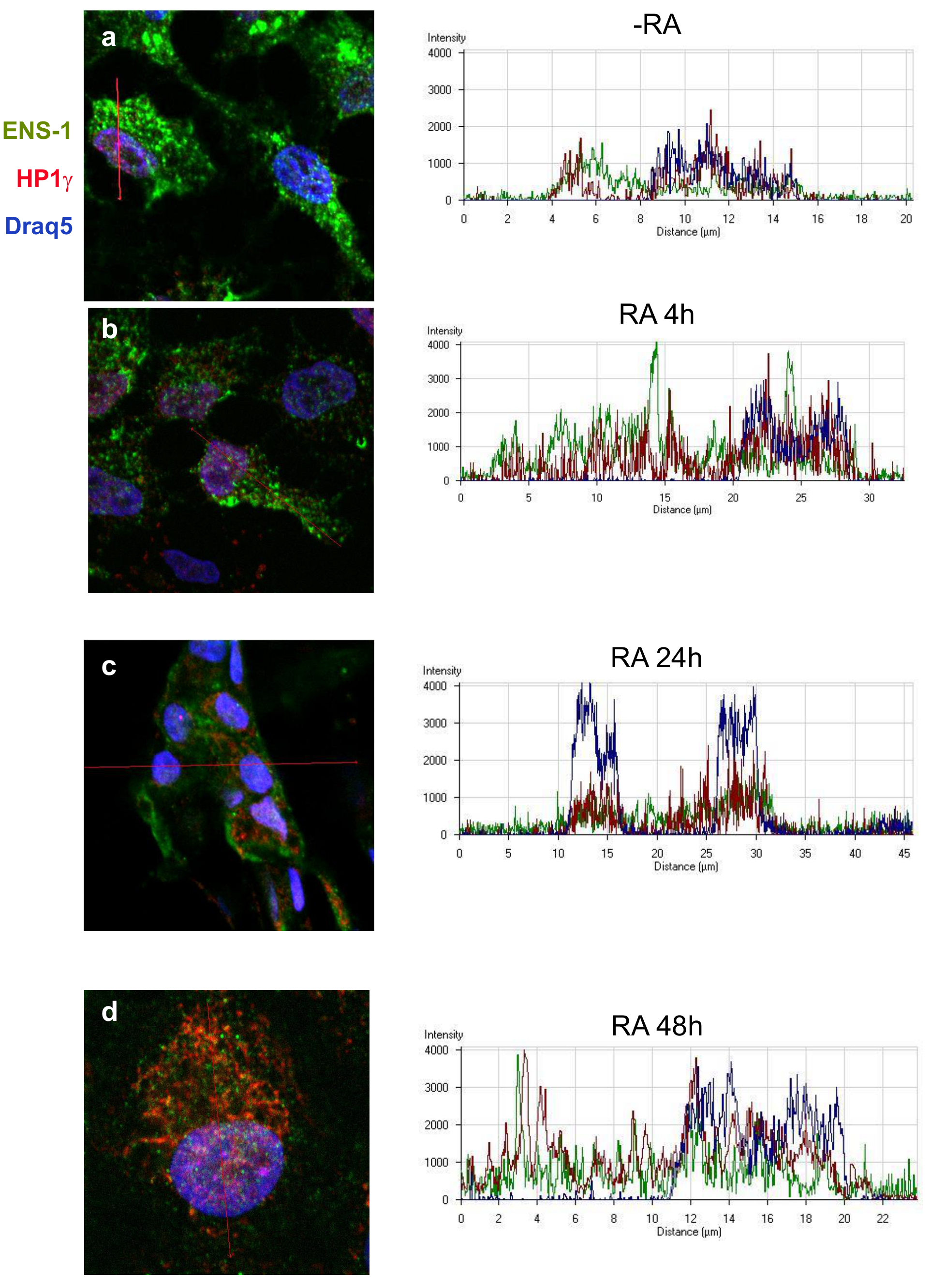

Supplement: Figure S2 — Distribution and colocalization of ENS-1 with HP1γ during differentiation of CES cells. Images are those presented in Figure 5C complemented with fluorescence intensity for the three labeling along the line represented in the merged image by the red arrow. CES cells (a) or cells differentiated for 4 h (b), 24 h (b) or 48 h (d) with retinoic acid were stained as in Figure 4 with anti-ENS-1 (green) and anti-HP1γ antibody (red). Signal intensities are presented as arbitrary units. (TIF) [file pone.0092039.s002.tif]

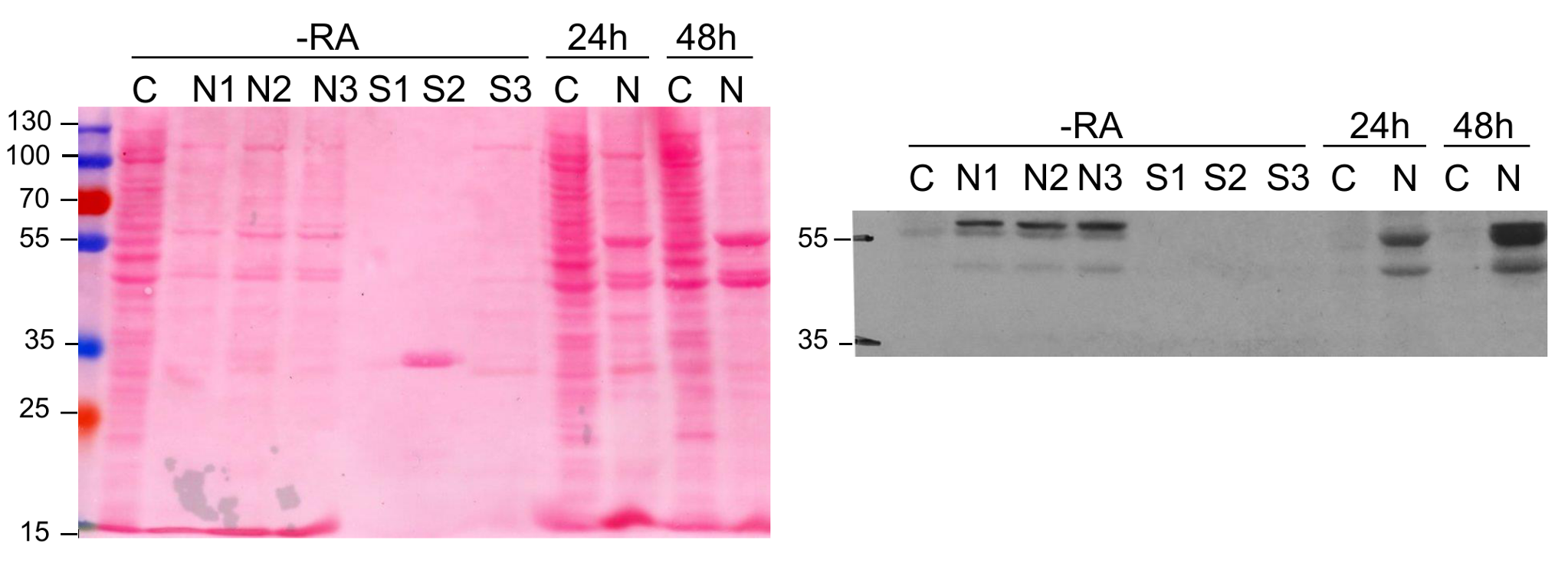

Supplement: Figure S3 — Western blot of soluble and insoluble protein fractions in the nucleus of CES cells. The whole blot corresponding to Fig. 5D is represented. N1 to N3 and S1 to S3 are loading replicates of respectively the insoluble and the soluble fractions of the nucleus. The S fractions had proteins concentrations lower (5 μg) than the N fractions (15 μg) but even in the S3 fraction that was slightly colored by Ponceau's red, no ENS-1 protein was detected. (TIF) [file pone.0092039.s003.tif]
